# Mechanisms of Uptake and Membrane Curvature Generation for the Internalization of Silica Nanoparticles by Cells

**DOI:** 10.1021/acs.nanolett.2c00537

**Published:** 2022-04-04

**Authors:** Valentina Francia, Catharina Reker-Smit, Anna Salvati

**Affiliations:** Department of Nanomedicine and Drug Targeting, Groningen Research Institute of Pharmacy, University of Groningen, A. Deusinglaan 1, 9713AV Groningen, The Netherlands

**Keywords:** Nanoparticle uptake, mechanism of endocytosis, membrane curvature, protein corona, nanomedicine

## Abstract

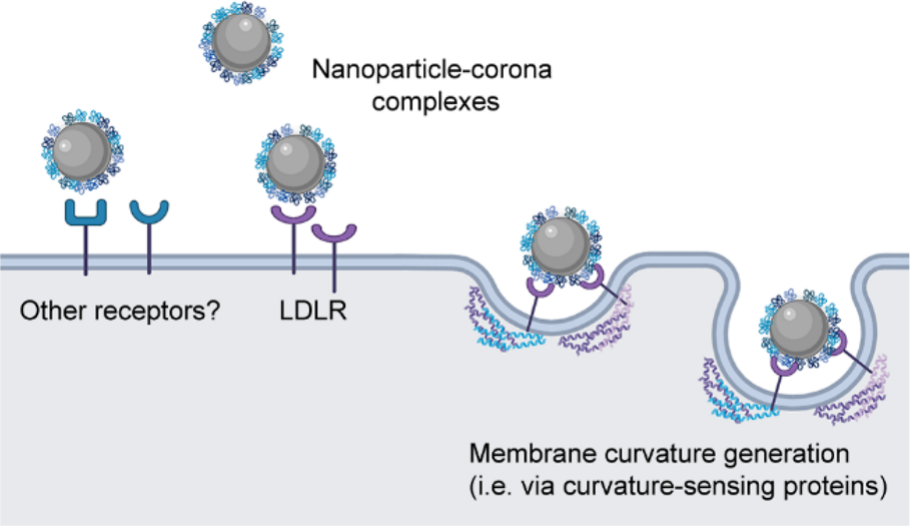

Nanosized drug carriers enter cells via active mechanisms of endocytosis but the pathways involved are often not clarified. Cells possess several mechanisms to generate membrane curvature during uptake. However, the mechanisms of membrane curvature generation for nanoparticle uptake have not been explored so far. Here, we combined different methods to characterize how silica nanoparticles with a human serum corona enter cells. In these conditions, silica nanoparticles are internalized via the LDL receptor (LDLR). We demonstrate that despite the interaction with LDLR, uptake is not clathrin-mediated, as usually observed for this receptor. Additionally, silencing the expression of different proteins involved in clathrin-independent mechanisms and several BAR-domain proteins known to generate membrane curvature strongly reduces nanoparticle uptake. Thus, nanosized objects targeted to specific receptors, such as here LDLR, can enter cells via different mechanisms than their endogenous ligands. Additionally, nanoparticles may trigger alternative mechanisms of membrane curvature generation for their internalization.

Nanosized drug carriers are used in nanomedicine to improve the delivery of drugs to their target.^1−3^ In order to deliver their drug payload, first they need to be recognized by cell receptors on the targeted cells, then they need to be internalized. Cell targeting can be achieved by decorating the nanocarrier surface with ligands capable of recognizing specific receptors on the targeted cells. However, once in a biological environment, for example, after intravenous administration, nanosized materials adsorb on their surface a biomolecule “corona”.^4−6^ In some cases, this layer can mask surface ligands, impairing targeting.^7^ At the same time, corona proteins themselves can be recognized by specific cell receptors, thus acting as a targeting moiety.^8−12^ Whichever the case, what drives nanoparticle internalization after the initial cell recognition is still unclear, and the details of the subsequent mechanisms of cellular uptake are often unknown. Many studies have tried to determine this,^13−17^ usually investigating the involvement of the major endocytic mechanisms, such as clathrin- and caveolin-mediated endocytosis and macropinocytosis, in nanoparticle uptake. However, it is hard to draw conclusions based on the results reported so far.^18−20^ This is due to multiple reasons, including the known limits of the different methods available to characterize the endocytosis mechanisms, the very different exposure conditions used in independent studies, thus differences in the nanoparticle corona, as well as the intrinsic complexity of endocytosis.^18,19,21−23^ The endocytosis field is still very active and research has shown that, next to the major uptake mechanisms, cells possess a variety of alternative mechanisms, often referred to as clathrin-independent endocytosis.^18,21−26^ Yet, the involvement of these alternative pathways in the uptake of nanosized drug carriers has rarely been investigated.

In all cases, in order to internalize extracellular materials, after the first interactions at the cell membrane and potential receptor interactions, cells activate different mechanisms of membrane curvature generation to bend the cell membrane and form an invagination in which the cargo is internalized.^22,26−28^ In clathrin-mediated endocytosis, membrane bending is achieved by the clustering of clathrin and a pool of other specialized proteins, while in the case of macropinocytosis actin-driven cell protrusions are formed to engulf the cargo. More recently multiple alternative mechanisms of curvature generation have been described.^22,28,29^ In many cases, membrane bending is mediated by proteins containing modules with curved structure, such as the so-called BAR (Bin/amphiphysin/Rvs) domains, which can recognize and induce membrane curvature.^28,29^ Yet, the mechanisms of membrane curvature generation involved in the uptake of nanosized materials have not been investigated so far. Computer simulations and *in vitro* studies with artificial membranes have shown that nanoparticles can induce several changes upon interaction with a lipid bilayer, for instance, by leading to sol–gel transitions in the lipid bilayer and impairing lipid lateral diffusion.^30,31^ The interactions of nanoparticles with a lipid bilayer depend on many factors, including, among others, nanoparticle size, the presence of a corona on the nanoparticles, as well as on the bilayer properties.^32,33^ Some studies have shown that nanoparticles can themselves induce membrane bending^34,35^ in ways similar to what observed with certain viruses of comparable sizes, capable of triggering their internalization.^26,36^ Thus, we hypothesized that by inducing changes in lipid bilayers and due to their capacity of bending membranes, nanoparticles themselves may be able to activate and assist membrane curvature generation for their endocytosis.

To test this, we have combined complementary techniques such as RNA interference (RNAi) and expression of nonfunctional mutants to characterize the mechanisms by which 50 nm silica nanoparticles (SiO_2_) with a human serum corona are internalized by cells. When coated with a corona formed in high concentration of human serum, more closely resembling *in vivo* conditions, these nanoparticles are recognized by the low density lipoprotein receptor, LDLR, via interactions mediated by their corona.^9,12^ Thus, these nanoparticles and corona conditions were chosen as a well characterized example to investigate the uptake mechanisms triggered by cells after the interaction of nanosized materials with specific cell receptors (in this case mediated by their corona). First, we tested whether the major endocytic pathways were involved and particularly clathrin-mediated endocytosis, as usually observed for the LDLR.^19,26^ Next, we tested the involvement of different proteins known to play a major role in clathrin-independent mechanisms. Finally, we studied whether a panel of BAR-domain proteins known to assist membrane curvature generation in different uptake pathways had a role in nanoparticle uptake.

As a first step, in order to form a corona promoting interaction with the LDLR,^9,12^ 50 nm silica nanoparticles were dispersed in ∼60 mg/mL human serum, and corona-coated nanoparticles were isolated from the unbound serum proteins to reduce their interference in the uptake process. Dynamic light scattering confirmed that homogeneous dispersions of corona-coated nanoparticles were obtained (Figure S1 shows an example of the results obtained for the corona-coated nanoparticles. We refer to Francia et al.^12^ for a more complete characterization of the same nanoparticles, both pristine and corona-coated.). Next, uptake was investigated in human epithelial cancer HeLa cells, here used as a model cell line commonly applied in many nanomedicine and endocytosis studies.^12,14,37,38^ Confocal fluorescence imaging confirmed nanoparticle uptake and accumulation in the lysosomes (Figure S2a). Uptake kinetics showed up to 70% uptake reduction in LDLR silenced cells, the effect being stronger at longer exposure times (Figure S2b). Thus, as expected, the uptake of the silica nanoparticles was mediated by the LDLR.

We then combined different methods to further characterize the mechanism of endocytosis. RNA interference (RNAi) was used to shut down the expression of a panel of proteins involved in different endocytic pathways (Figure 1a). This included the LDLR and the transferrin receptor (TFR), together with major markers of clathrin-mediated endocytosis (CLTC, CLTCL1, EPN1, DNM1-2), so-called caveolae-mediated endocytosis (CAV1, DNM1-2) (although debate on this mechanism and whether is used for nanoparticle uptake is still ongoing),^18,39^ and macropinocytosis (RAC1, ANKFY1, CDC42, ARF6). Next, we tested the role of proteins involved in different clathrin-independent mechanisms more recently characterized.^24,25^ These included flotillin-mediated (FLOT1, DNM2), CLIC/GEEC (CDC42, GRAF1), Arf6-mediated (ARF6), and RhoA-mediated (RHOA, RAC1, DNM1-2) endocytosis. We note that several of these markers are known to participate in multiple endocytic mechanisms, thus here as a first step we silenced their expression to determine their role in the uptake.

**Figure 1:**
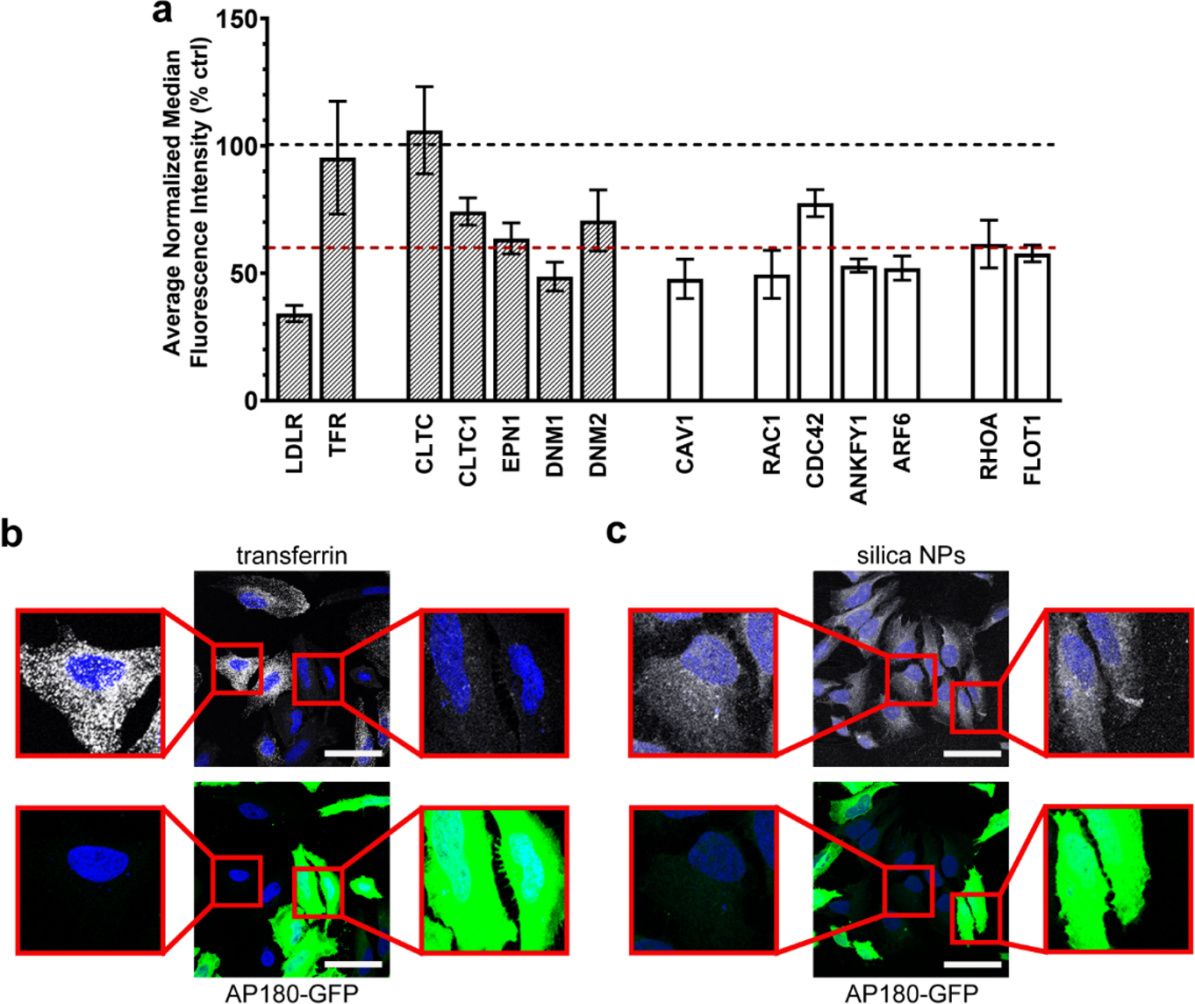
Characterization of uptake mechanisms of corona-coated SiO_2_ nanoparticles in HeLa cells. (a) HeLa cells were silenced for 72 h for a panel of endocytic targets (as indicated in the labels). Thus, cells were exposed for 14 h to 100 μg/mL corona-coated SiO_2_ nanoparticles prepared as described in Materials and Methods. Results are the average of the median cell fluorescence intensity measured by flow cytometry of three independent experiments, each performed with three replicate samples, normalized by the uptake in control cells silenced with a scramble siRNA. Error bars are the standard error of the mean. A black and a red dashed line at 100% and 60% uptake, respectively, are included as a reference (where 60% uptake is an indicative threshold on the effect of silencing on nanoparticle uptake). The results show uptake reduction in cells silenced for several of the selected targets. (b,c) Confocal fluorescence images of HeLa cells transfected with a plasmid carrying a GFP tagged AP180 whose expression blocks clathrin-mediated endocytosis. After 24 h, cells were exposed for 10 min to 15 μg/mL labeled transferrin in serum-free MEM or for 24 h to 100 μg/mL nanoparticle-corona complexes. Blue, DAPI stained nuclei; green, GFP expression of transfected cells; white, transferrin (b) or silica nanoparticles (c). Scale bar: 50 μm. Enlarged areas of the main panel for both nontransfected cells and transfected cells expressing GFP (green) are included on the left and right side of the images, respectively. The results confirm that in the cells expressing GFP-AP180 (green), where clathrin-mediated endocytosis is blocked, transferrin uptake is absent (b) but no effects are observed on nanoparticle uptake (c).

RT-qPCR confirmed that for most targets, the RNA levels after silencing were reduced by more than 90%, and in all cases by at least 70% (Figure S3a and Table S1 for primer details). Many of the proteins tested had a role in the uptake mechanism (Figure 1a), however uptake was the same after silencing the expression of clathrin heavy chain (CLTC). This suggested that despite the involvement of the LDLR uptake was not clathrin-mediated. Western blot analysis confirmed that clathrin was not detectable in the silenced cells (Figure S3b). In line with this, uptake of transferrin, known to enter cells via clathrin-mediated endocytosis, was reduced by 50 to 80% when silencing CLTC and other genes involved in this pathway (Figure S3c), confirming efficient inhibition in our conditions. To further rule out the involvement of clathrin-mediated endocytosis, additional studies were performed by overexpressing the C-terminal of the clathrin adaptor protein AP180. This is known to inhibit clathrin-mediated endocytosis.^40^ After transfection, the cells expressing GFP can be easily identified and in these cells clathrin-mediated endocytosis is blocked. Because of known variability in transfection efficiency across individual cells, cells that do not express GFP can be used as an internal control to determine the uptake when clathrin-mediated endocytosis is present. As expected, cells overexpressing C-term-AP180 (green) were not able to internalize transferrin (white, Figure 1b). Instead, nanoparticle uptake was the same in the transfected and nontransfected cells (Figure 1c). In line with these results, we previously showed that cell exposure to the pharmacological inhibitor chlorpromazine (CP), known to block clathrin-mediated endocytosis,^41^ had minor effects on SiO_2_ uptake with a reduction of only ∼30% after more than 3 h of exposure (here reproduced as a reference in Figure S4).^12^ Altogether, these results demonstrated that, despite the involvement of the LDLR, clathrin-mediated endocytosis was not the main pathway by which the cells internalized these nanoparticles.

On the contrary, other proteins involved in different uptake mechanisms had a role in nanoparticle uptake (Figure 1a). More in detail, a strong uptake reduction was observed after silencing dynamin expression (50% when silencing DNM1 and 40% when silencing DNM2). Dynamin is a key protein for clathrin-mediated endocytosis but also several clathrin-independent pathways are known to be dynamin-dependent.^18,25,26^ In line with the silencing results, we previously showed that inhibiting dynamin with dynasore^42^ reduced the uptake of ∼40% (also reproduced in Figure S4).^12^ These results suggested that, although not clathrin-mediated, uptake was dynamin-dependent.

Silencing CAV1, a gene involved in caveolae-mediated endocytosis, reduced SiO_2_ uptake by ∼50%, whereas silencing FLOT1, a marker for the flotillin-dependent pathway, reduced the uptake by ∼40%. Both pathways depend on the cholesterol present in the cell membrane. However, we previously showed that cholesterol depletion by methyl-beta cyclodextrin (MBCD) had only minor effects on nanoparticle uptake (also reproduced as a reference in Figure S4).^12^ A possible explanation for this discrepancy is that CAV1 and FLOT1 depletion indirectly affects nanoparticle internalization by a different mechanism, for instance, by decreasing membrane plasticity, since both are reported to be regulators of membrane tension.^43^

Silencing ARF6, a marker for ARF6-mediated endocytosis also involved in clathrin-mediated endocytosis and macropinocytosis,^18,25,26^ led to ∼50% uptake reduction. Next to this, silencing of targets involved in macropinocytosis, such as RAC1 and ANKFY1, reduced uptake by about 50%, while silencing CDC42 (also involved in phagocytosis) had only minor effects (∼20% uptake reduction). Several studies have suggested that macropinocytosis is a major pathway for the internalization of nanosized drug carriers.^17,44,45^ However, we previously showed that when exposing cells to EIPA, a selective inhibitor of macropinocytosis but also an inhibitor of RAC1 and CDC42 signaling,^46^ SiO_2_ uptake was reduced by only 20–30% after 6 h (also reproduced as a reference in Figure S4).^12^ RAC1 and ANKFY1 are involved in other mechanisms besides macropinocytosis. For example, RAC1 has a role in RhoA-mediated endocytosis, and silencing RHOA also reduced uptake by 40%, indicating that SiO_2_ uptake might depend on this pathway. Finally, we previously showed that blocking actin and microtubule polymerization led to up to 40% uptake reduction (also in Figure S4), suggesting an important role for actin and the cytoskeleton in the internalization mechanism.^12^

As additional controls, we measured LDLR expression and LDL uptake after silencing the different targets (Figures S3d-e). Silencing can alter the expression of associated pathways^18,19^ and a change in LDLR expression could indirectly affect SiO_2_ uptake, since these nanoparticles are internalized via this receptor. A mild increase in LDLR expression was found only in cells silenced for DNM1 and DNM2. Despite this, a clear nanoparticle uptake reduction was observed also in these silenced cells. On the contrary, LDLR expression was mildly reduced in cells silenced for RAC1, and this might contribute to the strong SiO_2_ uptake reduction observed in these cells.

Overall, these results suggested that several proteins involved in different uptake mechanisms seemed to have a role in the uptake of these nanoparticles, as opposed to one dominating pathway. Similar observations have been reported in other studies aiming at determining the mechanisms of nanoparticle uptake by cells.^12,14,17,20,47−49^ Alternatively, novel uptake mechanisms not yet characterized may be involved in the internalization of these special nanosized cargoes.

Thus, as a next step, we investigated the mechanism of membrane curvature generation involved in nanoparticle uptake. RNAi was used to silence the expression of a panel of BAR-domain proteins known to induce membrane curvature.^28,29,50,51^ RT-qPCR confirmed efficient silencing (in most cases higher than 80%, except for BIN2 for which only 40% reduction was observed, see Figure S5a for details).

Interestingly, silencing the expression of several BAR domain proteins caused a marked reduction of nanoparticle uptake (Figure 2a). The strongest effects were observed after silencing BIN1 (∼60% uptake reduction). Western blot analysis confirmed that BIN1 expression was effectively depleted after silencing (Figure S5b). Uptake kinetics by flow cytometry and uptake quantification by fluorescence imaging further confirmed a reduction in nanoparticle uptake in BIN1-silenced cells (Figure 2 a-c). Amphiphysin2, which is encoded by BIN-1, is one of the most studied BAR domain proteins. It is an N-BAR protein able to recognize and induce membrane bending.^52,53^ Its strong effect on nanoparticle uptake is particularly interesting considering that BIN1 is mainly known to be involved in clathrin-mediated endocytosis, while our results clearly demonstrated that the uptake of these nanoparticles is not clathrin-mediated, despite LDLR involvement (Figure 1).

**Figure 2:**
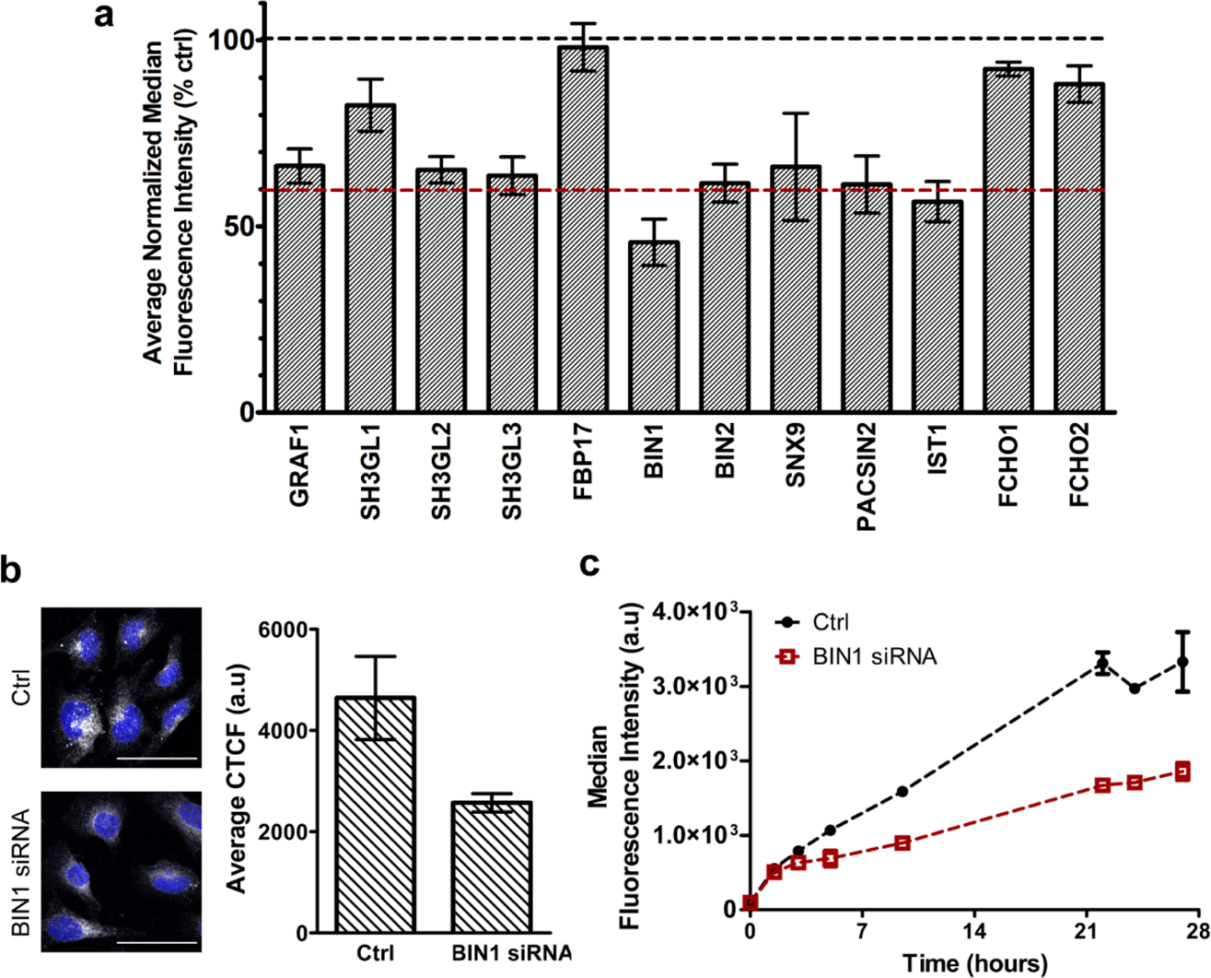
Role of curvature sensing proteins in the uptake of corona-coated SiO_2_ nanoparticles in HeLa cells. (a) HeLa cells were silenced for 72 h for a panel of BAR domain curvature sensing proteins (as indicated in the labels). Thus, cells were exposed for 14 h to 100 μg/mL corona-coated SiO_2_ nanoparticles formed as described in the Materials and Methods. The results are the average of the median cell fluorescence intensity measured by flow cytometry over four independent experiments, each performed with three replicate samples, normalized by the uptake in control cells silenced with a scramble siRNA. Error bars are the standard error of the mean. A black and a red dashed line at 100% and 60% uptake, respectively, are included as a reference (where 60% uptake is an indicative threshold on the effect of silencing on nanoparticle uptake). The results show uptake reduction in cells silenced for several targets. (b) Confocal fluorescence images and corresponding intensity quantification of HeLa cells silenced for BIN1 and exposed for 14 h to 100 μg/mL corona-coated SiO_2_ nanoparticles. The results are the average corrected total cell fluorescence (CTCF) obtained from at least 4 images for each condition (see Materials and Methods for details) and confirm strong uptake reduction in BIN1 silenced cells. Scale bars 50 μm. Blue: DAPI for nuclei. (c) Uptake kinetics of corona-coated SiO_2_ nanoparticles in BIN1 silenced HeLa cells (BIN1 siRNA, red line) or control cells silenced with a scramble siRNA (Ctrl, black line). The results are the average and standard deviation over three replicate samples of the median cell fluorescence intensity obtained by flow cytometry.

Together with BIN1, silencing the expression of GRAF1, SH3GL2, SH3GL3, BIN2, SNX9, PACSIN2, and IST1 reduced uptake by ∼40% as well. In order to exclude indirect effects of silencing, also in this case LDLR expression and LDL uptake were determined after silencing the expression of each of the target genes. Silencing IST1 reduced LDLR expression by 30% and consequently also LDL uptake (Figure S6a-b). This can indirectly explain the observed SiO_2_ uptake reduction after IST1 silencing. Only in cells silenced for FCHO2 an increase in LDLR expression was observed, accompanied by increased LDL uptake. However, nanoparticle uptake was not affected in these cells. On the contrary, silencing SH3GL2, SH3GL3, SNX9, and PACSIN2 had minor or no effects on LDLR expression, though it decreased SiO_2_ uptake by around 40%, suggesting that these targets have a specific role in nanoparticle uptake.

Similar studies were performed on lung epithelial A549 cells (Figure S7). We previously showed that the uptake of SiO_2_ nanoparticles with a human serum corona formed at high serum concentration is mediated by the LDLR also in these cells.^12^ As expected, silencing the LDLR reduced uptake by ∼60%. Interestingly, also in A549 cells, SiO_2_ uptake decreased by ∼40% after silencing BIN1 (∼30% after silencing BIN2), PACSIN2, and IST1.

These results, although preliminary, confirmed that BAR-domain curvature sensing proteins do have a role in nanoparticle uptake and their involvement may vary in different cell types, since different cells may express a different pool of this class of proteins. A better understanding of the activity of curvature sensing proteins in different cells may open up new ways to target and promote nanoparticle uptake in specific cells.

BAR domain proteins can sense and recognize lipid membranes with a different curvature.^22,28,29^ Thus, their activity is likely to vary depending on the curvature generated by the cargo, as well as the curvature of the cargo itself. Larger nanoparticles have a smaller curvature, thus nanoparticles of different sizes offer the unprecedented opportunity to test how the activation and involvement of curvature sensing proteins change with cargo curvature. To this end, we exposed HeLa cells to human serum-coated silica nanoparticles of 50, 100, and 200 nm after silencing the expression of the different curvature sensing proteins for which an effect was observed (Figure 2). The uptake was mediated by the LDLR only in the case of the smaller particles (Figure 3). Interestingly, in all cases the effect of silencing on the uptake was smaller when increasing nanoparticle size, thus when decreasing cargo curvature. This suggested that the activity of these proteins depends on the curvature of the cargo, here varied by changing nanoparticle size.

**Figure 3:**
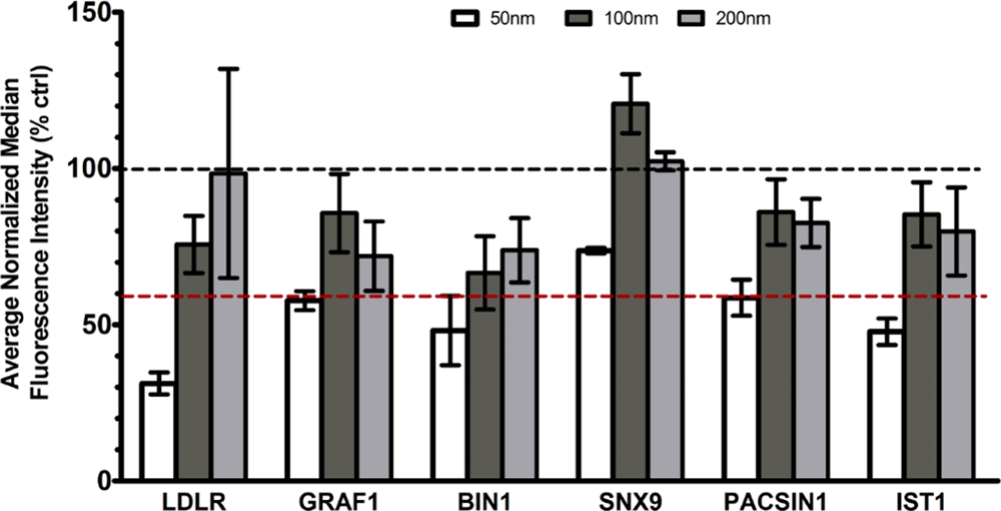
Role of LDLR and curvature sensing proteins in the uptake of 50, 100, and 200 SiO_2_ NP-corona complexes in HeLa cells. HeLa cells were silenced for 72 h for a panel of BAR domain curvature sensing proteins (as indicated in the labels). Thus, cells were exposed for 14 h to 100 μg/mL corona-coated SiO_2_ nanoparticles prepared as described in the Materials and Methods. The results are the average and standard error of the median cell fluorescence intensity measured by flow cytometry over three independent experiments, each performed with 2–3 replicate samples, normalized by the uptake in control cells silenced with a scramble siRNA. A black and a red dashed line at 100% and 60% uptake, respectively, are included as a reference (where 60% uptake is an indicative threshold on the effect of silencing on nanoparticle uptake).

Altogether, our results showed that despite the observed role of the LDLR and several reports suggesting the involvement of clathrin-mediated endocytosis in nanoparticle uptake,^14,54,55^ the uptake of 50 nm SiO_2_ nanoparticles is not clathrin-mediated. This apparent discrepancy is likely due to the fact that nanoparticle uptake has been rarely studied in the presence of high human serum concentration, as we did here. Indeed, we previously found that the uptake of these same 50 nm SiO_2_ nanoparticles is clathrin-mediated when measured in low serum conditions, as typically used for *in vitro* studies.^12^ It is important for the field to carefully consider the conditions used for these types of studies in order to take into account effects related to exposure conditions and nanoparticle corona formation.^56^

Interestingly, our results also showed that silencing the expression of several proteins involved in different endocytic pathways affected nanoparticle uptake, leading to ∼40–60% uptake reduction (Figure 1a). This may be connected to known limits of the methods used to study the uptake mechanisms and cross-talk between pathways.^18,19,57^ However, such observations also support the hypothesis that multiple pathways may be triggered within the same cells, as opposed to one dominating pathway, likely via interaction with different receptors, next to the LDLR.^12^ At the same time, alternative pathways may be involved. Indeed, our results showed that many BAR domain proteins known to sense and induce membrane curvature do have a role in nanoparticle uptake (Figure 2) and their involvement varies with nanoparticle curvature (Figure 3). Thus, as illustrated in Figure 4, after the initial recognition by the LDLR (likely also other receptors), nanoparticles may trigger their internalization via alternative mechanisms of curvature generation involving a pool of these specialized curvature sensing proteins.

**Figure 4:**
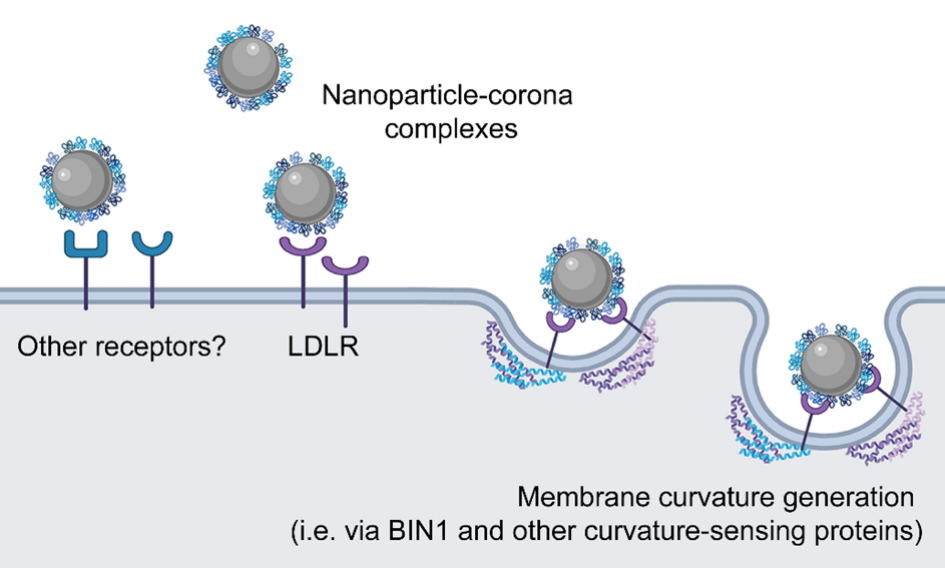
Proposed alternative mechanism of membrane curvature generation and nanoparticle uptake. The nanoparticle-corona complexes interact with the LDLR and possibly other receptors and induce membrane bending. Thus, curvature sensing proteins, such as BIN1, capable to sense membrane curvature, are activated and assist membrane curvature generation for nanoparticle internalization. Although further research is necessary to fully demonstrate the proposed mechanism and characterize the role of the identified curvature sensing proteins in nanoparticle uptake, similar mechanisms have been described for the uptake of other nanosized cargoes, such as viruses.^26,36^ Image created with BioRender.com.

Further studies are required to test how these proteins participate in the uptake mechanism and whether they are recruited to the cell membrane upon membrane bending. Similarly, it is important to understand how the interaction with specific receptors is coupled to the subsequent mechanism of uptake, thus the mechanism of membrane curvature generation which cells activate for nanoparticle internalization.

Overall, the results presented suggest that nanosized cargoes may assist nanoparticle uptake in alternative ways via activation of curvature-sensing proteins, capable of inducing membrane curvature. Similar mechanisms have been described for other natural nanosized cargoes such as viruses,^26,36^ indicating that nanosized materials, whether natural or man-made, may share important similarities in the way they are processed by cells. Understanding the molecular details of the mechanisms of membrane curvature generation for nanoparticle uptake will help to clarify how to design targeted nanomedicines with an improved efficacy.

## Supporting Information Available

Detailed materials and methods, nanoparticle characterization by dynamic light scattering, quantification of mRNA levels of different markers and LDLR after silencing their expression and additional control experiments in silenced cells, role of curvature sensing proteins in nanoparticle uptake in a different cell line (A549) (PDF)
