# Supplementary material for: Mechanisms of Uptake and Membrane Curvature Generation for the Internalization of Silica Nanoparticles by Cells
Source: Nano Lett. 2022 Apr 4;22(7):3118–24. doi: 10.1021/acs.nanolett.2c00537 (PMC9011393; doi:10.1021/acs.nanolett.2c00537)
Supplement: Supplementary file 1 — nl2c00537_si_001.pdf [file nl2c00537_si_001.pdf]

Supporting Information

Mechanisms of Uptake and Membrane Curvature Generation for the Internalization of Silica Nanoparticles by Cells

*By Valentina Francia, Catharina Reker-Smit, Anna Salvati**

Department of Nanomedicine & Drug Targeting, Groningen Research Institute of Pharmacy, University of Groningen, A. Deusinglaan 1, 9713AV Groningen, the Netherlands

Materials and Methods

Cell culture

HeLa cells CCL-2 purchased from ATCC (Manassas, VA, USA) were cultured in complete cell culture medium (cMEM) which consisted of MEM (Gibco Thermofisher Scientific, Landsmeer, Netherlands) supplemented with 10% v/v Fetal Bovine Serum (FBS, Gibco Thermofisher Scientific) and grown at 37 °C, 5% CO_2. The cells were routinely tested to exclude mycoplasma contamination and were kept in culture for up to maximum 20 passages after defrosting.

Nanoparticle characterization and corona complexes preparation

Red fluorescently labelled 50 nm silica nanoparticles were purchased from Kisker Biotech, Steinfurt, Germany (maximum excitation wavelength of 569 nm and maximum emission of 585 nm). In order to prepare and isolate corona-coated nanoparticle complexes at a high serum content, more closely resembling blood protein content (60-80 mg/ml), 300 µg/ml nanoparticles were dispersed in 62 mg/ml human serum from pooled donors (6.2% w/V in grams per 100 mL solution) (TCS BioSciences Ltd Botolph Claydon, UK) for 1h at 37°C and then centrifuged at 16100 rcf for 1h. The pellet was resuspended in PBS and then diluted in serum-free MEM to a final concentration of 100 µg/ml of nanoparticles, roughly corresponding to 7,64 x 10^11 nanoparticles/ml (with a silica density of 2 g/cm^3). Dynamic Light Scattering (DLS) was used to characterize the corona-coated nanoparticles prepared as described above using a Malvern Zetasizer Nano ZS (Malvern Instruments Ltd., Worcestershire, UK). The results of 3 repeated measurements of 5 runs each are shown.

Pharmacological Inhibitor studies

The mechanisms of uptake of 50 nm SiO_2 nanoparticles were characterized using inhibitors of endocytosis and previously optimized protocols to exclude toxicity.[1, 2] Briefly, HeLa cells were seeded at a concentration of 50000 cells/cm^2 in a 24-well plate (Greiner Bio-One BV, A. Alphen on den Rijn, Netherlands). 24h after seeding, HeLa cells were pre-incubated for 10 (or 20 minutes for Nocodazole) in serum-free MEM culture medium with the different inhibitors at the following concentrations: 5-(N-Ethyl-N-isopropyl)amiloride (EIPA) 100µM, chlorpromazine hydrochloride (CP) 10 µg/ml, methyl-β-cyclodextrin (MβCD) 2.5 mg/ml, dynasore 25 µg/ml (all from Sigma-Aldrich St. Luis, USA), nocodazole 5 µM (Biovision, California, USA), cytochalasin D 2.5 µg/ml (Thermofisher Scientific). Afterwards, cells were exposed to 100 µg/ml nanoparticle-corona complexes dispersed in serum-free MEM in standard conditions or in the presence of the inhibitors. Controls were performed for each experiment, as reported elsewhere.[2] Samples were collected and prepared for flow cytometry as described below.

RNA interference

RNA interference was used to shut down the expression of different proteins involved in endocytosis and curvature sensing proteins. Briefly, 13000 cells/cm^2 were seeded in a 24-well plate (Greiner Bio-One BV). 24h after seeding, HeLa cells were washed in serum-free MEM for 20 minutes and then each well was incubated with 250 µl of a mix composed of 45 µl oligofectamine (Thermofisher), 10nmol of siRNA and Opti-MEM (Thermofisher). In order to exclude eventual effects of the transfection reagents and procedure on cells and on nanoparticle uptake, nanoparticle uptake in the silenced cells was compared to the uptake in control cells treated with oligofectamine and a scramble RNA using the same procedures (negative control). After 4 hours, 125 µl of MEM supplemented with 30% v/v Fetal Bovine Serum was added to cells. 72h after silencing, cells were incubated with 100 µg/ml nanoparticle-corona complexes dispersed in serum-free MEM and prepared as described above for the indicated times. Alternatively, cells were incubated for 4 hours with 1 or 2 µg/ml of BODIPY- or Dil-labelled LDL (ThermoFisher Scientific), or for 10 minutes with 15 µg/ml of fluorescently labeled human transferrin (Alexa546-TF, ThermoFisher Scientific) in serum-free MEM. Samples were collected and analyzed by flow cytometry or fluorescence imaging as described below.

Plasmid transfection with AP180

The construct containing the GFP-tagged C-terminal of Ap180 expressed under a constitutive promoter was kindly provided by Yvonne Vallis and Harvey T McMahon (Cambridge University, UK).[3] HeLa cells were transfected with 0.2 ng of plasmid DNA, using 0.6 µl Fugene (Promega) as transfection reagent in cMEM. After 24h, cells were washed in serum-free MEM and incubated with nanoparticle-corona complexes or transferrin, as described above. Cells were then washed and samples prepared for fluorescence imaging as described below.

Flow Cytometry Analysis

Flow cytometry was used to measure the fluorescence intensity of cells incubated with fluorescent 50 nm SiO_2 nanoparticles, LDL, or transferrin. After the required exposure time, cells were washed once with cMEM and twice with PBS in order to reduce the presence of nanoparticles and markers adhering to the cell membrane. HeLa cells were harvested using 0.05% trypsin–EDTA for 5 min at 37 °C, collected, centrifuged for 3 min at 300 rcf, and resuspended in 100 µl PBS for the measurement. Cell fluorescence was recorded using a BD FACSarray (BD Biosciences, Erembodegem, Belgium) using a 532 nm laser for SiO_2 nanoparticles and Dil-LDL, or a Cytoflex Flow S Cytometer (Beckman Coulter, Woerden, Netherlands) with a 561 nm laser for SiO_2 nanoparticles and for Alexa546 transferrin, and 488 nm for BODIPY LDL. Data were analyzed using Flowjo data analysis software (Flowjo, LLC). Cell debris and cell doublets were excluded by setting gates in the forward and side scattering double scatter plots. A total of at least 20000 cells were acquired per sample and each sample was performed in triplicate (unless specified). Then the average and standard deviation of the median cell fluorescence intensity were calculated. Experiments were repeated at least 3 times to confirm reproducibility. The results are the average and standard error of the average results obtained in 3 independent experiments (unless specified).

Immunohistochemistry

For immunohistochemistry, Hela cells were plated on glass coverslips inserted in 24-well plates and experiments were performed as described above. Cells were incubated with nanoparticle-corona complexes or transferrin. Lysosomal staining was performed by incubating cells with a mouse primary antibody against LAMP-1 (clone H4A3, BD Biosciences) for 1 h, followed by 1 h incubation with an Alexa Fluor 488 goat secondary anti-mouse antibody. After each step of antibody incubation, cells were washed 3 times with PBS. Nuclei were stained with 0.2 µg/ml

DAPI (4',6-diamidino-2-phenylindole) and glass slides were mounted with Mowiol 4-88 mounting medium (EMD Chemical, Inc, CA, USA). Image acquisition was performed using a Leica TCS SP8 fluorescent confocal microscope (Leica Microsystems, Wetzlar, Germany) with a 405 nm laser for DAPI excitation, a 488 nm laser for Alexa Fluor®488 or GFP, and a 552 nm laser for 50 nm SiO_2 nanoparticles or transferrin. Images were processed using ImageJ software (http://www.fiji.sc). In order to compare nanoparticle uptake in BIN1 silenced cells and control cells silenced with a scramble siRNA, the corrected total cell fluorescence (CTCF) was calculated. Individual cells were selected manually from at least 4 different images for each condition and their integrated density and area were extracted. For each image, areas without cells were selected in order to obtain the mean fluorescence of the background. Thus, for each image, the CTCF was calculated as the integrated density of the selected cells minus their cell area multiplied by the mean fluorescence of the background. The results are the average corrected total cell fluorescence obtained from at least 4 frames for each condition for a total of 38 cells for control cells and 19 cells for BIN1 silenced cells.

mRNA expression

The expression levels of silenced proteins were determined by RT-qPCR using the primers listed in Table S1. 72 h after transfection, the cells recovered from 4 wells were merged and their total mRNA was isolated using an Invitrap Spin Cell RNA Mini Kit (Stratec Molecular GmbH, Berlin, Germany) according to the instruction provided by the manufacturer. Reverse transcription of 2 µg mRNA into cDNA was performed using a Reverse Transcription System (Promega, Leiden, The Netherlands) in an Eppendorf Mastercycler gradient (the following cycle was used: 20 ˚C for 10 min, 42 ˚C for 30 min, 20 ˚C for 12 min, 99 ˚C for 5 min and 20 ˚C for 5 min). The transcription levels were measured by quantitative real time PCR (SensiMix™ SYBR kit, Bioline, Taunton, MA) in a ABI7900HT sequence detection system (Applied Biosystems, Foster City, CA) from cDNA (20 ng per sample). The Ct values were obtained using SDS 2.4 software (Applied Biosystems). For each target, 3 or 4 replicate wells were prepared (as specified in the captions) and the average Ct value and its standard deviation were calculated. Results are expressed as fold-change of the averaged Ct values of negative control (Neg) related to Ct values of the sample investigated (S) as follows:

$$\text{Fold change} = 2^{-(\text{Mean Neg} - \text{Mean S})} \qquad (1)$$

Gene	Forward (left)	Reverse (right)
LDLR	GTGACAATGTCTCACCAAGCTC	CACGCTACTGGGCTTCTTCT
TFRC	TGAAGAGAAAGTTGTCGGAGAAA	CAGCCTCACGAGGGACATA
CLTC	TGAGAAAAGAAGAAGAACAAGCTACA	ACACTGGGTCCTGCTGTCA
CLTCL1	TTTATTAATGGACCCAACAATGC	CATTCCCTCCTCGTAACAGC
DNM1	AGGGCTTTATGTCGAGCAAG	[illegible]
DNM2	CATCAACACGAACCATGAGG	CTTGTTCAGCTGCGTGCTC
EPN1	GAGAGCAAGAGGGAGACTGG	AAGACGTCAGCAAGGTCCAT
CAV1	ACAGCCCAGGGAAACCTC	GATGGGAACGGTGTAGAGATG
RAC1	CTGATCAGTTACACAACCAATGC	CATTGGCAGAATAATTGTCAAAGA
CDC42	CATCGGAATATGTACCGACTGTT	TGCAGTATCAAAAAGTCCAAGAGTA
ANKFY1	AAACTAGCAAATCGGTTTCAGC	GAGACATAACACCCTTCTCACATC
ARF6	TGAACACAAAGTTGCTAGATGCT	TGCTGTGTTTCCCCCATC
RHOA	GGAGCTAGCCAAGATGAAGC	GCCAATCCTGTTTGCCATA
FLOT1	ATTCTAACTCGCCTGCCAGA	GCATCTGTGAGGGCTGAAG
GRAF1	CAGGCACGGTCTTCGATAA	GCCAGTCTTTCCGTTCAGAG
SH3GL1	GGGGCTGAAGAAGCAGTTC	TTGCTGGTGACATCCACCT
SH3GL2	AGGCACTCCCAAACCTTCA	CAGGTTCAAAGTCGTACAGAGC
SH3GL3	GCAGAAATTCTTTCAAAAACCACT	CCTCGGATCTTCGACACAGT
FBP17	CAGCAGCACATCGAGACTTG	GCGTTCAAATCGCCTTTTAC
BIN1	CGTGCAGAAGAAGCTCACC	TGCTCATCCTTGGTCTCATCT
BIN2	TTGAACAAAGCGCTAGCAAC	CTTCAGGTCCTTGTACAGCTTG
PACSIN2	CCGGGCCCTGTATGACTAT	TCCTCCATCTTGGTCAGCTC
IST1	CTTAAACTATTGGAGAAAAAGAAAAC G	CCCAGCAGCCAGATAGTC
FCHO1	TGAAACGCCATTCTTCACG	TCTGACTGCAATCTCTCTGCAC
FCHO2	GATACAAAAATTTGCTGAGTCAAAAG	TGTCACATTCTTCAAATTCAATGAG

Table S1. Primers used in this study for RT-qPCR. RT-qPCR was performed as described in the Methods to determine the expression levels of some cell receptors (LDL and transferrin receptor), and a series of targets involved in different endocytic pathways and curvature sensing proteins.

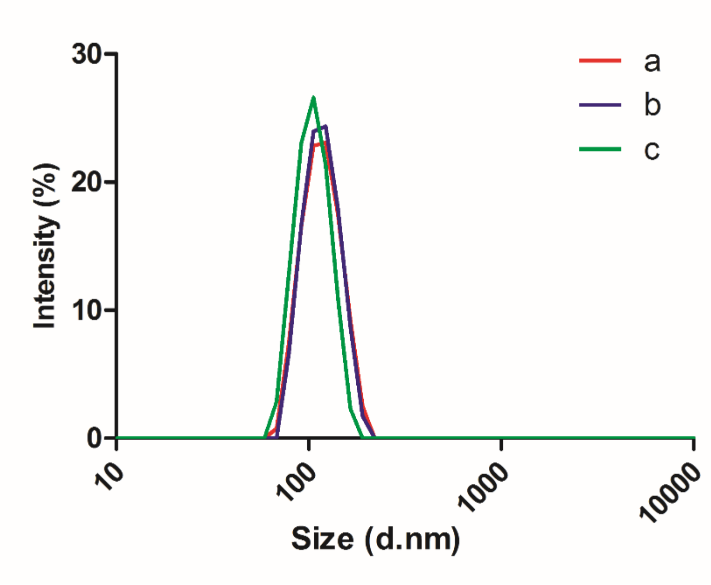

Figure S1. Size distribution by intensity (diameter, d.nm) of corona-coated 50 nm silica nanoparticles. Briefly, 300 µg/ml SiO_2 nanoparticles were incubated for 1 hour at 37˚C in 60 mg/ml human serum, centrifuged for 1 hour and resuspended in MEM to 100 µg/ml prior to characterization by Dynamic Light Scattering (DLS). The results show that corona-coated SiO_2 nanoparticles could be resuspended and formed homogenous dispersions. Red, blue and green curves are the results of three different size measurements of the same sample. A more complete characterization of these same nanoparticles, pristine and corona-coated, can be found in Francia et al.[2]

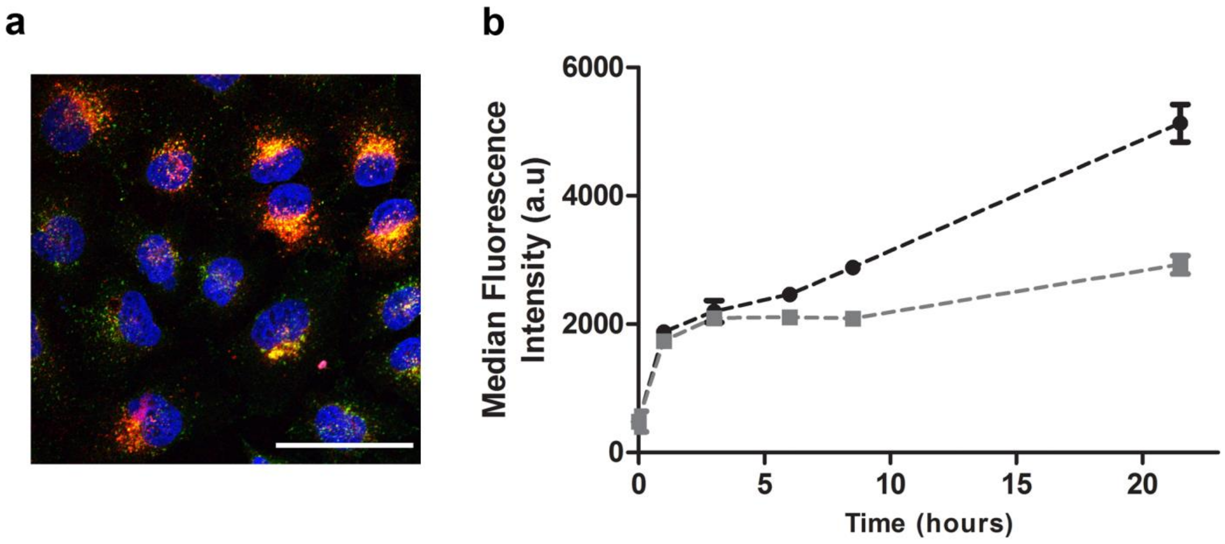

Figure S2. Nanoparticle intracellular location and uptake kinetics. Corona-coated SiO_2 nanoparticles were prepared by incubating 300 μg/ml 50 nm silica nanoparticles in human serum at a concentration of 60 mg/ml. Nanoparticle-corona complexes were then isolated by centrifugation and incubated at a final concentration of 100 μg/ml of nanoparticles on HeLa cells. a) Confocal fluorescence images of HeLa cells exposed for 14 hours to corona-coated SiO_2 nanoparticles (red). Blue: DAPI for nuclear staining, green: LAMP1 for lysosome staining. Scale bar 50 μm. Microscopy confirmed nanoparticle uptake and accumulation in the lysosomes. b) Uptake in silenced cells for the LDLR (gray line) or control cells silenced for a scramble siRNA (black line). The results are the average and standard deviation over 3 replicates of the cell fluorescence intensity obtained by flow cytometry. Uptake was reduced in cells silenced for LDLR, the effect being more evident at longer exposure times.

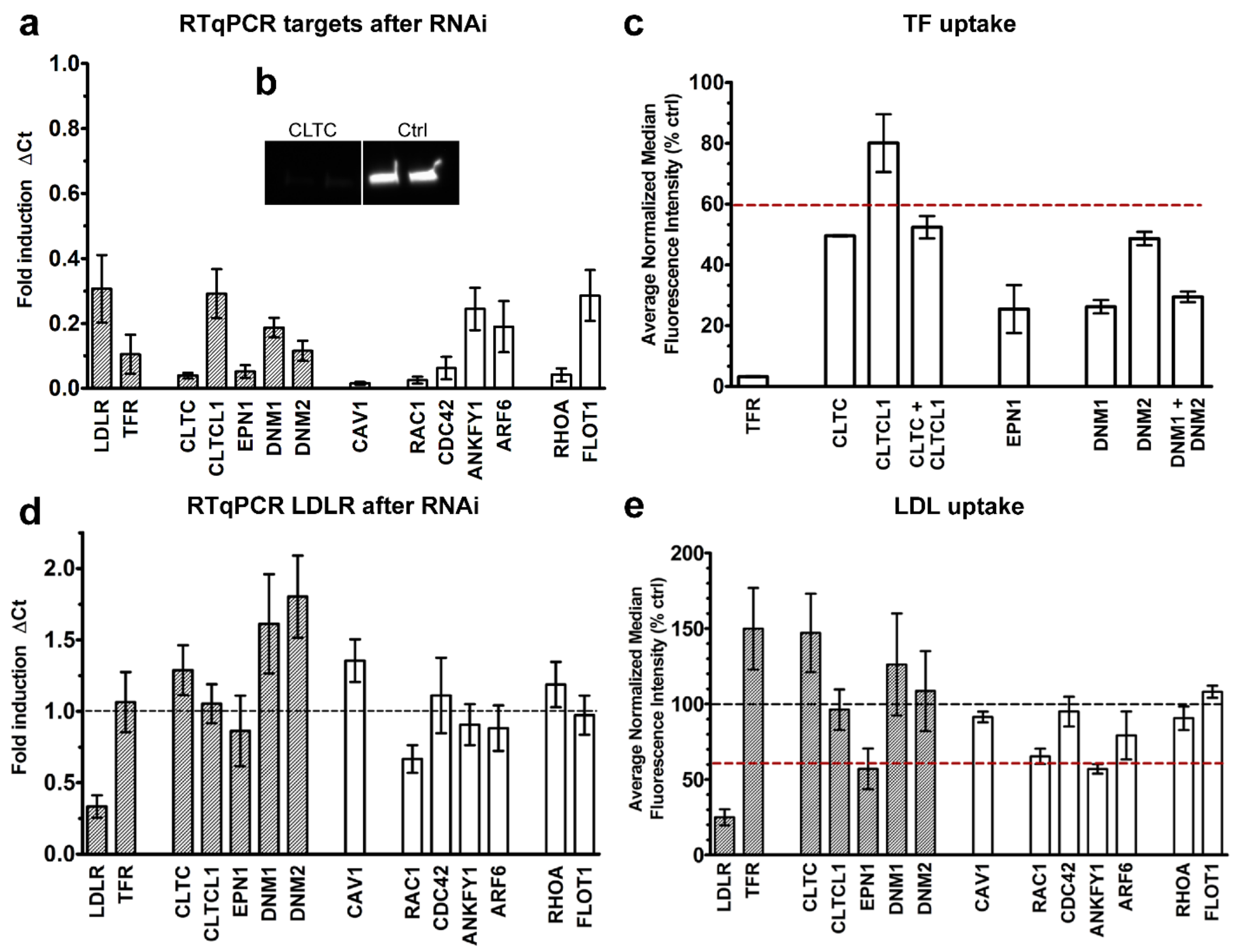

Figure S3. Silencing controls for a panel of endocytosis targets. a) Expression levels of endocytic targets in silenced HeLa cells. HeLa cells were silenced as described in the Methods for reducing the expression of the selected targets (as defined in the labels) or with a control scramble siRNA. After 72 h silencing, RTqPCR was used to quantify their expression in silenced cells. Results are expressed as fold change of silenced cells in comparison to cells silenced with a scramble siRNA, calculated from Ct values. Data are the average of 3 independent experiments (2 for CLTCL1), each performed with 3 replicate samples, and the error bars represent the standard error of the mean. The results confirm the reduction of the expression levels of all of the targets investigated in the silenced cells. b) Western blot of clathrin heavy chain (CLTC) in HeLa cells silenced with a siRNA for CLTC or with a scramble siRNA (ctrl). CLTC protein is not detectable by western blot, confirming the efficacy of CLTC silencing. c) Uptake of labeled transferrin (TF) in silenced cells. HeLa cells silenced for the targets indicated were incubated for 10 min with 15 μg/ml fluorescently labelled transferrin in serum-free MEM. Cell fluorescence was measured by flow cytometry and

expressed as percentage compared to control cells treated with a scramble siRNA. Error bars represent the standard error of the mean over 3 independent experiments, each performed with 3 replicate samples. A red dashed line at 60% uptake is included as a reference (where 60% uptake is an indicative threshold on the effect of silencing on nanoparticle uptake). The results confirm more than 90% transferrin uptake reduction in cells silenced for the transferrin receptor (TFR), around 70% uptake reduction in cells silenced for EPN1 and DNM1 and up to 50% for CLTC and DNM2. d) Expression levels of LDLR in HeLa cells silenced for a panel of endocytosis targets. HeLa cells were silenced for the targets indicated. RTqPCR was used to quantify the expression levels of LDLR in silenced cells in order to monitor potential indirect effects of the silencing on LDLR expression. Results are represented as fold change of silenced cells compared to cells silenced with a scramble siRNA, calculated from Ct values as described in the Methods. Data are the average of 3 independent experiments, each performed with 3 replicate samples, and error bars represent the standard error of the mean. The results suggest that silencing of DNM1 and DNM2 causes an increase in LDLR expression, while silencing of RAC1 decreases LDLR expression. e) Uptake of LDL in silenced cells. HeLa cells silenced for the targets indicated were incubated for 4 hours with 1-2 µg/ml BODIPY- or Dil-LDL in serum-free MEM. The results are the average and standard deviation of three independent experiments and represent the cell fluorescence measured by flow cytometry of silenced cells normalized by the cell fluorescence of control cells treated with a scramble siRNA. A black and a red dashed lines at 100% and 60% uptake, respectively, are included as a reference (where 60% uptake is an indicative threshold on the effect of silencing on nanoparticle uptake). The results suggest that, while silencing the LDLR caused a very strong reduction of LDL uptake, silencing some of the clathrin-mediated endocytosis targets (CLTC, CLTCL1, DNM1, and DNM2) did not reduce LDL uptake. For some of these targets, this may be explained by the increased LDLR expression in cells silenced for these targets observed in panel d.

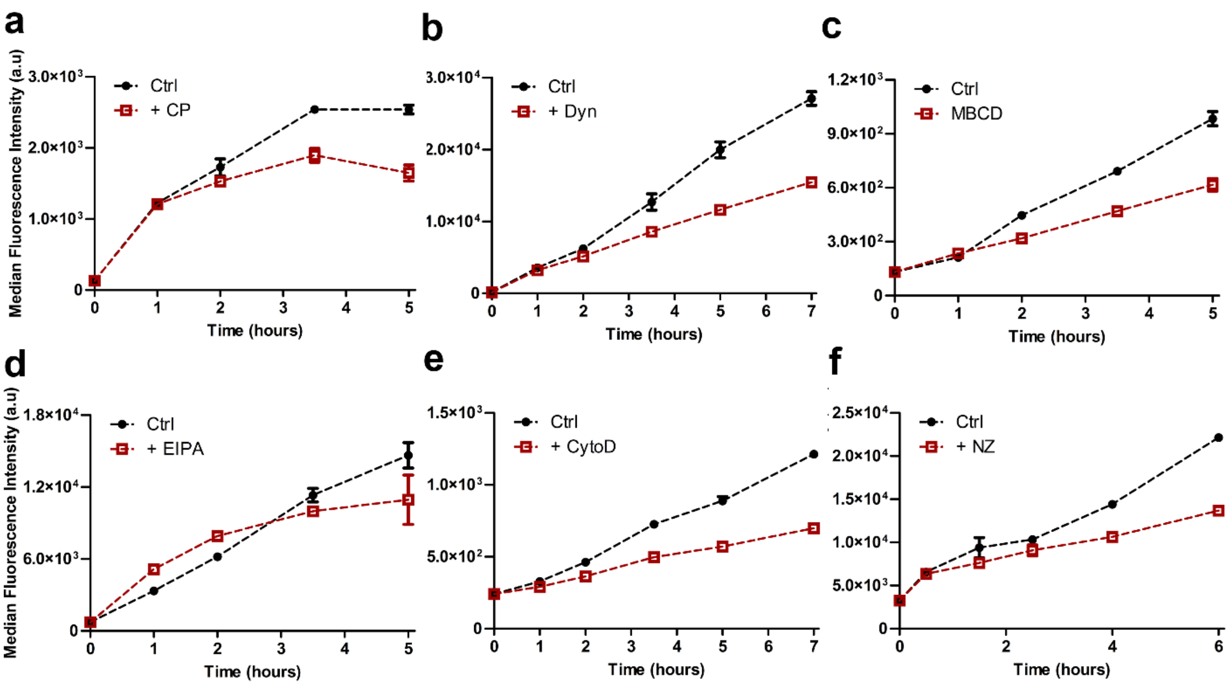

Figure S4. Uptake kinetics of corona-coated nanoparticles in HeLa cells treated with a panel of transport inhibitors. HeLa cells were exposed to 100 µg/ml corona-coated SiO_2 nanoparticles in standard conditions (Ctrl) or in the presence of 10 µg/ml chlorpromazine (CP), 100µM 5-(N-Ethyl-N-isopropyl) amiloride (EIPA), 2.5 mg/ml methyl-β-cyclodextrin (MBCD), 25 µg/ml dynasore (Dyn), 5 µM nocodazole (NZ) or 2.5 µg/ml cytochalasin D (CytoD). Results are the average and standard deviation of the median cell fluorescence intensity measured by flow cytometry over 3 technical replicates. All these results are reproduced as a reference from Francia et al. *ACS Nano* **2019,** *13* (10), 11107-11121.[2] Copyright 2019 American Chemical Society. As discussed in more detail elsewhere,[1, 4, 5] CP and EIPA are commonly used to block clathrin-mediated endocytosis and macropinocytosis, respectively. MBCD sequesters cholesterol from the cell membrane, thus it is used to test whether uptake is cholesterol-dependent (multiple pathways depend on cholesterol in the cell membrane. We refer to Iversen et al[4] for a more complete description of this and of the effects of cholesterol depletion in cells). Dynasore is a dynamin inhibitor commonly used to test whether uptake is dynamin-dependent (this includes clathrin-mediated endocytosis and other clathrin-independent pathways).[6] Finally cytochalasin and nocodazole are used to block the polymerization of actin and tubulin, thus to test their involvement in uptake. Pathways that depend on actin and tubulin include clathrin-mediated endocytosis, macropinocytosis, and different clathrin-independent pathways.[1, 5]

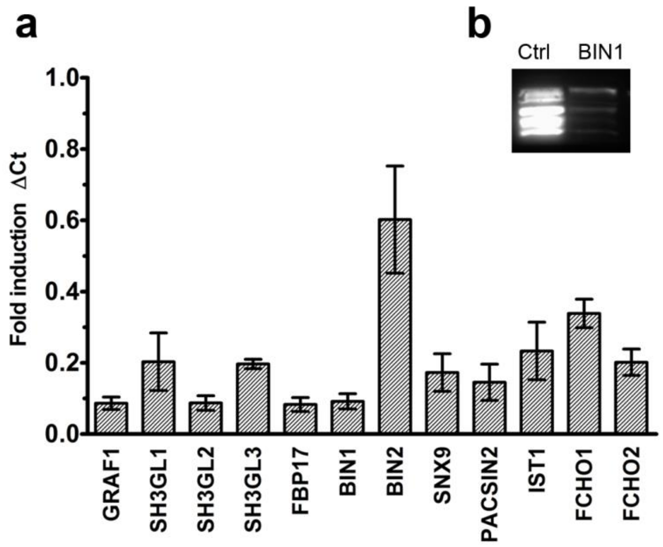

Figure S5. Silencing controls for a panel of BAR domain curvature sensing proteins. a) Expression levels of BAR domain proteins in silenced HeLa cells. HeLa cells were silenced as described in the Methods for reducing the expression of a series of proteins (as defined in the labels) or with a control scramble siRNA. After 72 h silencing, RTqPCR was used to quantify the expression of the different targets in the silenced cells. Results are the fold increase of silenced cells in comparison to cells silenced with a scramble siRNA, calculated from Ct values as described in the Methods. Data are the average of 3 independent experiments (2 for FCHO1), each with 4 replicates per condition, and the error bars are the standard error of the mean. b) Western blot of BIN1 in HeLa cells silenced for BIN1 and with a scramble siRNA (ctrl). RT-PCR confirms an excellent reduction of expression for all silenced targets, except for BIN2, probably due to its low expression in HeLa cells (high Ct). (The results for this target are anyway included, given the strong nanoparticle uptake reduction observed in BIN2 silenced cells) For BIN1, the western blot confirms the reduction of BIN1 also at the protein level in BIN1 silenced cells.

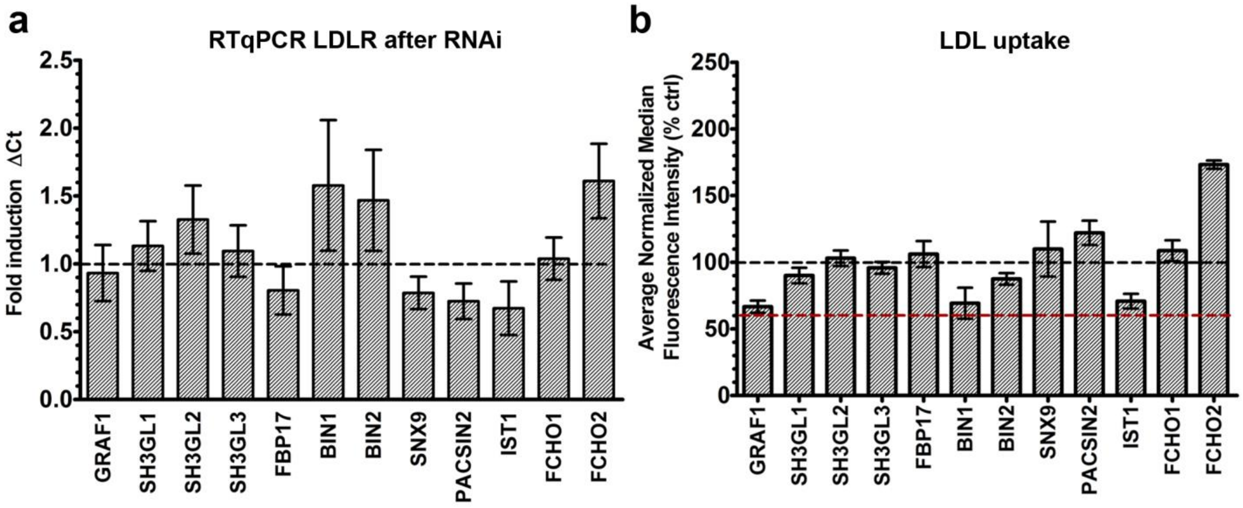

Figure S6. Additional controls in cells silenced for a panel of BAR domain curvature sensing proteins. a) Expression levels of LDLR in HeLa cells silenced for a panel of BAR domain curvature sensing proteins. HeLa cells were silenced for the targets indicated. RTqPCR was used to quantify the expression levels of LDLR in cells silenced for each target in order to monitor silencing effects on LDLR expression. Results are the fold increase of silenced cells in comparison to cells silenced with a scramble siRNA, calculated from Ct values as described in the Methods. Data are the average of 3 independent experiments, each with 4 replicates, and error bars represent the standard error of the mean. The results suggested only minor effects on LDLR expression, with a mild increase in cells silenced for BIN1, BIN2 and FCHO2. b) Uptake of LDL in silenced cells. HeLa cells silenced for the targets indicated were incubated for 4 hours with 1-2 µg/ml fluorescently labelled BODIPY-LDL in serum-free MEM. The results are the average and standard error of the mean over 3 independent experiments, each performed with 3 replicate samples, of the cell fluorescence measured by flow cytometry, normalized by the uptake in control cells treated with a scramble siRNA. A black and a red dashed lines at 100% and 60% uptake, respectively, are included as a reference (where 60% uptake is an indicative threshold on the effect of silencing on nanoparticle uptake). The results show increased uptake in cells silenced for FCHO2, which can be explained by the increased LDLR expression in these cells. Importantly, LDL uptake was lower in cells silenced for GRAF1, BIN1, and IST1 suggesting a potential role for these targets in LDL uptake. The reduced LDL uptake in IST1 silenced cells might be due to the reduction in LDLR expression in these cells.

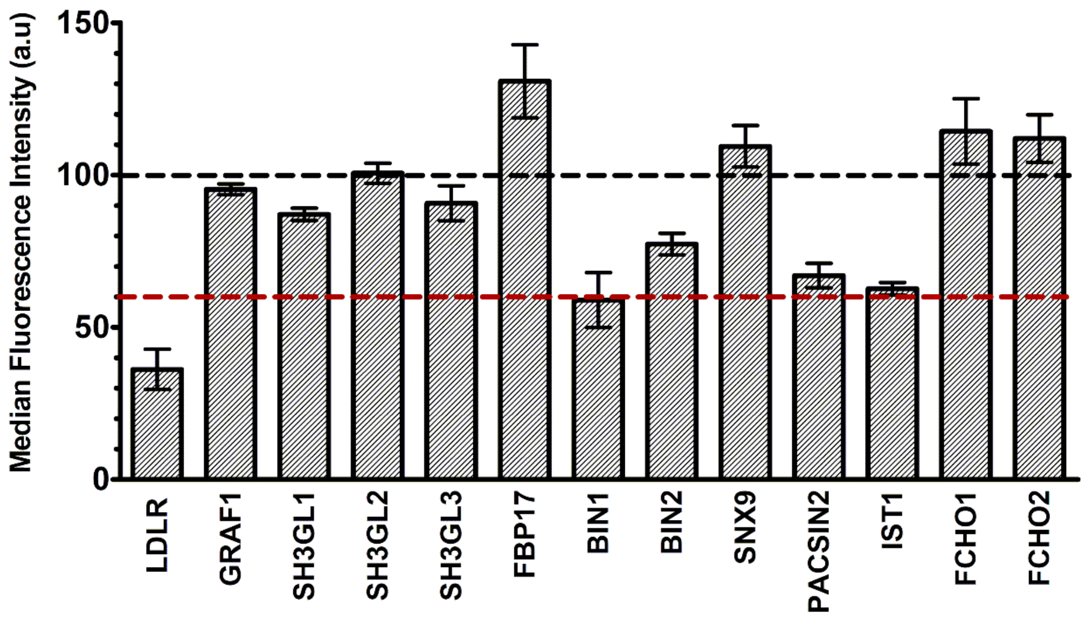

Figure S7. Role of curvature sensing proteins in the uptake of corona-coated SiO_2 nanoparticles in A549 cells. A549 were silenced for 72 hours for a panel of BAR domain curvature sensing proteins (as indicated in the labels). Thus, cells were exposed for 14-16 hours to 100 µg/ml corona-coated SiO_2 nanoparticles formed as described in the Methods. The results are the average of the median cell fluorescence intensity measured by flow cytometry over 3 independent experiments, each performed with 3 replicate samples, normalized by the uptake in control cells silenced with a scramble siRNA. Error bars represent the standard error of the mean. A black and a red dashed lines at 100% and 60% uptake, respectively, are included as a reference (where 60% uptake is an indicative threshold on the effect of silencing on nanoparticle uptake). The results show that also in these cells, silencing the LDLR strongly reduced nanoparticle uptake. Uptake reduction was observed also in cells silenced for BIN1 (and to a smaller extent its isoform BIN2), PACSIN2 and IST1, as observed in HeLa cells. However, for other targets for which an effect was observed in HeLa cells, no effects were observed in A549 cells. These results indicate that curvature sensing proteins do play a role in nanoparticle uptake also in these cells, but different types are involved depending on the cell type.

References

1. Francia, V.; Reker-Smit, C.; Boel, G.; Salvati, A., Limits and challenges in using transport inhibitors to characterize how nano-sized drug carriers enter cells. *Nanomedicine (Lond)* **2019,** *14* (12), 1533-1549.

2. Francia, V.; Yang, K.; Deville, S.; Reker-Smit, C.; Nelissen, I.; Salvati, A., Corona Composition Can Affect the Mechanisms Cells Use to Internalize Nanoparticles. *ACS Nano* **2019,** *13* (10), 11107-11121.

3. Ford, M. G.; Pearse, B. M.; Higgins, M. K.; Vallis, Y.; Owen, D. J.; Gibson, A.; Hopkins, C. R.; Evans, P. R.; McMahon, H. T., Simultaneous binding of PtdIns(4,5)P2 and clathrin by AP180 in the nucleation of clathrin lattices on membranes. *Science* **2001,** *291* (5506), 1051-5.

4. Iversen, T.-G.; Skotland, T.; Sandvig, K., Endocytosis and intracellular transport of nanoparticles: Present knowledge and need for future studies. *Nano Today* **2011,** *6* (2), 176-185.

5. Rennick, J. J.; Johnston, A. P. R.; Parton, R. G., Key principles and methods for studying the endocytosis of biological and nanoparticle therapeutics. *Nature Nanotechnology* **2021,** *16* (3), 266-276.

6. Mayor, S.; Pagano, R. E., Pathways of clathrin-independent endocytosis. *Nat Rev Mol Cell Biol* **2007,** *8* (8), 603-612.
